# Aberrant *AHRR*, *ADAMTS2* and *FAM184* DNA Methylation: Candidate Biomarkers in the Oral Rinse of Heavy Smokers

**DOI:** 10.3390/biomedicines11071797

**Published:** 2023-06-23

**Authors:** Hernán Guillermo Hernández, Gloria Cristina Aranzazu-Moya, Efraín Hernando Pinzón-Reyes

**Affiliations:** 1 School of Dentistry, Universidad Santo Tomás, Bucaramanga 680001, Colombia; 2 PhD Program in Dentistry, Universidad Santo Tomás, Bucaramanga 680001, Colombia; 3 Facultad de Ciencias Médicas y de la Salud, Instituto de Investigación Masira, Universidad de Santander, Bucaramanga 680003, Colombia; ehpinzon@udes.edu.co

**Keywords:** DNA methylation, smoking, biomarkers, epigenomics, mouth mucosa

## Abstract

Objective. To identify DNA methylation patterns of heavy smokers in oral rinse samples. Methods. Genome-wide DNA methylation data was imported from Gene Expression Omnibus *GSE70977* using the GEOquery package. Two independent sets were analyzed: (a) 71 epigenomes of cancer-free subjects (heavy smokers *n* = 37 vs. non-smokers *n* = 31); for concordance assessment (b) 139 oral-cancer patients’ epigenomes (heavy smokers *n* = 92 vs. non-smokers *n* = 47). Differential DNA methylation for CpG positions and at the regional level was determined using Limma and DMRcate Bioconductor packages. The linear model included sex, age, and alcohol consumption. The statistical threshold was set to *p* < 0.05. Functional gene prioritization analysis was performed for gene-targeted analysis. Results. In individuals without cancer and heavy smokers, the *FAM184B* gene was found with two CpG positions differentially hypermethylated (*p* = 0.012 after FDR adjustment), in a region of 48 bp with an absolute methylation difference >10% between groups (*p* = 1.76 × 10^−8^). In the analysis corresponding to oral-cancer patients, we found *AHRR* differentially hypomethylated cancer patients, but also in subjects without oral cancer in the targeted analyses. Remarkably, *ADAMTS2* was found differentially hypermethylated in heavy smokers without a diagnosis of cancer in two consecutive probes cg05575921 (*p* = 3.13 × 10^−7^) and cg10208897 (*p* = 1.36 × 10^−5^). Conclusions: Differentially methylated *AHRR,* ADAMTS2, and *FAM184B* genes are biomarker candidates in oral rinse samples.

## 1. Introduction

Tobacco smoke is one of the biggest risk factors for the disease of the oral mucosa caused by different mechanisms, including the induction of an increased number of Langerhans cells and greater induction of aldo-keto reductases, enzymes linked to genotoxicity [1]. In vitro studies report some regulatory dose-dependent changes in genes as *CYP1A1*, *CYP1B1,* and *AHRR* at the transcriptional level [2].

Tobacco smoke has been related to different oral diseases, such as periodontal disease, and some potentially malignant disorders, such as actinic cheilitis, nicotine stomatitis, and oral cancer. Different studies around the world related smoking and oral cancer, with an odds ratio (OR) of 6.19 (95% CI: 3.83–10.00) in Italy [3], 5.45 (95% CI: 2.74–10.85) in Brazil [3], and 14.64 (95% CI: 10.2–21.1) in India [4].

The pack-year index measures smoking intensity defined as the number of packs of cigarettes smoked per day multiplied by the number of years the person has smoked [5]. In heavy smokers, a high pack-year index increases cancer risk and associated mortality compared to non-smokers and heavy non-smokers [6,7]. Additionally, heavy smokers have a worse response to oral cancer treatment [8]. Smoking is known to affect the oral microbiome, with high levels of Gram-negative organism colonization in the tongue, a site with high rates of malignant transformation. Smoking is also the strongest factor in increasing microbial acetaldehyde production and reducing total salivary antioxidant capacity [9,10].

In this context, some risk factors can favor tumor promoter gene expression or inhibit the antitumor immune response, modifying DNA methylation at specific CpG sites in determined loci. If these specific loci are identified in risk factors, such as smoking, screening strategies with specific biomarkers could be established. The ultimate goal of discovering specific biomarkers is to facilitate early diagnosis, risk identification, targeted prevention, the follow-up of proposed treatments, and prognosis to reduce morbidity and mortality [11,12,13,14]. While some studies have been conducted to elucidate the carcinogenic effects of tobacco smoke on the bronchial epithelium and other diseases, the epigenetic profile of buccal cells [15] or DNA methylation differences in cancer [16], the epigenetic effect underlying environmental risk factors remains poorly understood. Accessible biomarkers of heavy smoking in oral tissue demand more research. Interestingly, a study in oral and pharyngeal cancer made epigenome data from 223 oral rinse samples available with the code GSE70977 in the Gene Expression Omnibus repository [16]. These data have been exclusively used to study patients with oral cancer, without determining the differential methylation patterns in oral rinse samples from cancer-free or cancer-diagnosed smokers compared to nonsmokers or studying differentially methylated genomic patterns at the regional level. The use of data from public repositories enables analyses that answer relevant questions while rationalizing resources [17] and contribute to understanding harmful phenotypic effects and to the progress of personalized medicine as a future guideline in the staging, management, prevention, and subclassification of the different neoplasms of the stomatognathic system [18]. Therefore, secondary bioinformatic analyses using available datasets, performing a functional genetic prioritization analysis guided by the results of positional and regional differential methylation analyses, could help to elucidate the clinical differences in oral mucosa diseases related to smoking.

The main objective of this work was to evaluate the buccal rinse cell genome-wide DNA methylation signature in heavy smokers using available Illumina DNA methylation 450 K BeadChip data from oral rinse-derived DNA. A secondary objective was to direct the evaluation of DNA methylation in heavy smokers, restricting the analysis to oral cancer-related genes selected by a gene prioritization bioinformatic approach. Therefore, this work is proposed as a secondary bioinformatic analysis, relevant to oral epigenetics and dentistry, to study the epigenome of smoking habits on the Illumina HumanMethylation450 BeadChip platform by identifying differentially methylated genomic sites and regions and addressing the knowledge gap about epigenetic patterns in cells collected from oral rinse in patients with a smoking habit, which are relevant in multiple oral diseases [19,20].

## 2. Materials and Methods

### 2.1. Available Data (Population)

We evaluated available data from 223 DNA methylation epigenomes assessed by the Infinium Illumina 450 K platform corresponding to buccal rinse cells primarily used in a cancer study with different aims and analyses [21]. Data were imported from the public dataset GSE70977, freely available in the Gene Expression Omnibus for secondary analyses [22].

The accessible population corresponded to 223 epigenomic profiles from buccal rinses collected at different hospitals in Boston, MA, USA [21]. Seventy-four patients were nonsmokers, and 149 patients were heavy smokers. The present study aimed to compare the DNA methylation profiles of heavy smokers in separate subsets (with or without cancer). However, a supplementary analysis including both subsets may be provided on reasonable request.

For each subset of the genome datasets, with and without a cancer diagnosis, we performed a separate analysis to compare heavy smokers with the respective control group [23]. For each subset, the corresponding heavy smoker group included individuals with a pack-year smoking history index >10, e.g., one daily cigarette pack for 10 years (1 × 10) or half daily cigarette pack for 20 years (0.5 × 20) [5]; for each control group, the epigenomic profiles from subjects with a pack-year smoking index less than 1 were selected. Considering the objective of the present study, all analyses were performed in a stratified form for each subset, avoiding mixing patients of different cancer statuses. The data subset of noncancer subjects had 71 epigenomes. The data subset of cancer subjects diagnosed with head and neck cancer had 152 epigenomes. We evaluated for inclusion all the data available in dataset GSE70977. Therefore, no sample size calculation was performed; however, adequate power was found in the Results section.

### 2.2. Data Importation, Cleansing, and Quality Control

DNA methylation, clinical and demographic data were imported from the GSE70977 dataset using the GEOquery R Bioconductor package [24] for the corresponding statistical and bioinformatics analyses. The dataset content consistency was manually visualized and cross-checked through verification data importation/verification R scripts for both the phenotypic and epigenetic data. Quality control processes were performed, including the corresponding fluorescence detection validation. Only data with a detection *p* value of less than 0.0000001 compared to the background signal passed this QC step. As a result, both samples and probes with more than 2% were excluded. In addition, QC included bimodal pattern density plot verification of beta values through the *minfi* package. For this purpose, we used the beta values normalized via the preprocess *Funnorm* of the *minfi* package available in the GEO dataset [25].

### 2.3. Statistical and Bioinformatic Analyses

This study used R Bioconductor, a statistical and bioinformatics platform to favor the reproducibility of bioinformatic analyses, based on an open-source policy, which allowed the design of scripts for all relevant analyses [26]. The statistical analysis was performed using the Limma package to compare differential methylation positions (DMPs) and differentially methylated regions (DMRs) in heavy smokers and nonsmokers as described below.

### 2.4. Genome-Wide Differential Methylation Positions Analysis for Heavy Smokers

Genome-wide DNA methylation was analyzed at the CpG position level to detect differentially methylated positions (DMPs) by modeling the study variable (heavy smokers or controls) and covariables, i.e., age, sex, and alcohol consumption, using robust methods. The delta of the beta filter for DMP was set at 0.09. The statistical significance threshold after FDR correction applied to genome-wide analysis was set at *p* < 0.05. Subsequently, gene assignment to identified DMPs was performed, considering a 2 kb distance from each probe to the nearby transcription start site (TSS associated with the gene), as previously used elsewhere [27,28].

### 2.5. Genome-Wide Differentially Methylated Regions Analysis for Heavy Smokers

In addition, we performed a differentially methylated region (DMR) analysis using the DMRcate package based on the Limma package [29] with code built in-house. We previously used to add a ‘delta of beta’ filter set to >6% [28].

### 2.6. Gene Prioritization for Analysis Focused on Specific Genes

Endeavour Bioinformatics tools facilitate the identification of promising candidate genes related to a condition or disease, based on training genes previously identified as associated with a condition or disease and curated annotations bioinformatic datasets (e.g., ‘Gene Ontology’, ‘InterPro’, ‘Biomolecular pathways’ such as ’Reactome’ gene interaction and protein interaction networks, e.g., ‘BioGrid’, and ’IntAct’, between others. The list of bets candidates is determined by their connections with the training genes [28,30]. This strategy allows complementation of the initial result using a list of genes in a directed analysis, as has been performed in previous studies [31]. Here, we used a combination of a results-driven approach, and a direct evaluation of candidate genes was performed using a prioritization analysis through the Endeavour Bioinformatic tool [28,30].

### 2.7. Endeavour Parameters Were Set As Follows:

(a) Input 1 (list of training): genes identified in the nondirected analysis (*AHRR*, *ADAMTS2*, *FAM184B*) and with an OMIM assignation for “Orolaryngeal cancer” (*CDKN2A*).
(b) Input 2 (list of candidates): genes evaluated/annotated by Infinium Illumina DNA Methylation 450 K.
(c) For the gene prioritization analysis, the statistical threshold for identification was *p* < 0.01; otherwise, the settings in Endeavour were set to default [32].

### 2.8. Analyses of Differentially Methylated Positions (DMPs) and Regions (DMRs) of Specific Loci

The DMP and DMR analyses were performed only for the genes identified in the gene prioritization analysis using the same procedure as the untargeted analysis. The prerequisites for DMPs in this targeted analysis were a limit of significance *p* value < 0.005 and a minimum DNA methylation difference between groups of 6%.

### 2.9. Effect of Cell Composition Sensitivity Analysis

To address a potential confounding factor due to cell composition, we used a recently available data [33], considering cell composition heterogeneity as a confounding variable in a sensitivity analysis using the estimateLC function from ewastools r-package in conjunction with BeadSorted.Saliva.EPIC Bioconductor package [34]. The results of these cell estimations of leucocytes vs. epithelial cells were included in the final model using Limma Bioconductor Package with the same parameters described above.

## 3. Results

Of the 71 available noncancer methylomes, 68 were included for analysis to identify DNA methylation differences associated with heavy smokers (HSs). Three methylomes were excluded by applying the selection criteria to the set of subjects without cancer diagnosis (two samples) and excluding such with poor quality findings in the quality control (QC) step (one sample). Of the 68 noncancer methylomes analyzed, 37 belonged to heavy smokers, and 31 belonged to controls.

**Figure 1:**
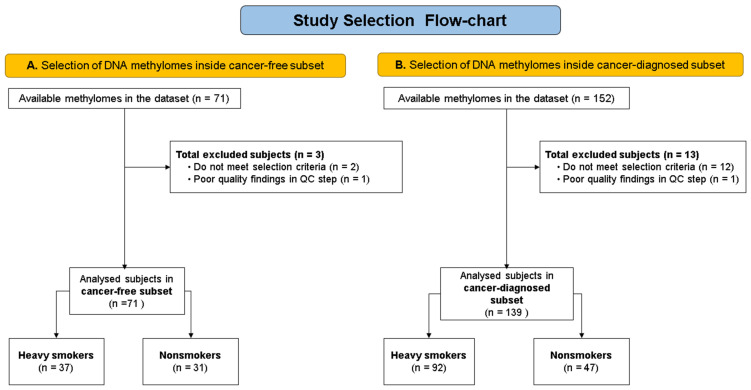
Study selection flowchart. A. Selection of DNA methylomes for the heavy smoker and control groups, in the cancer-free subset. B. Selection of DNA methylomes for the heavy smoker and control groups, in the cancer-diagnosed subset.

No significant differences in age or gender were found between heavy smokers and nonsmokers in the subset of patients without cancer (Figure 1).

However, differences were found between smokers and nonsmokers regarding alcohol consumption; therefore, this variable was included in the model to eliminate its effect on differential DNA methylation results. Demographic data are shown by subset for each group of interest (heavy smokers vs. nonsmokers) (Table 1).

Of the initial subset of 152 available methylomes corresponding to subjects with cancer, 139 were finally included for the analyses to identify DNA methylation differences associated with heavy smokers (HSs). Twelve were excluded by applying the selection criteria, and one more was ruled out due to poor-quality findings in the quality control (QC) step. Of the 139 subjects in this subset, 92 were heavy smokers and 47 were nonsmokers (Figure 1). No differences were found regarding age and gender. However, regarding alcohol consumption, significant differences were found between smokers and nonsmokers in the cancer patient subset (Table 1); therefore, for this analysis, the alcohol variable was also included in the model to eliminate its effect on differential methylation results.

In the noncancer subset, the genome-wide hypothesis-free analysis showed differentially hypermethylated positions in the *ADAMTS2* and *FAM184B* genes in the initial model (Table 1) and the final model including alcohol consumption (Table 2). Two positions were found in the final model that considered alcohol consumption (Table S1).

In the cancer-diagnosed subset, the genome-wide hypothesis-free analysis showed four differentially hypomethylated positions, one in the *AHRR* gene and three in intergenic regions (Table 3). Two of these positions were also found after applying the final model including alcohol consumption (Table S2).

In the noncancer subset, DMR analysis found 18 regions supported by more than one differential CpG for smokers compared to nonsmokers in the initial model. Among them is the gene *FAM184B* (Table S3). In the final model, DMR analysis showed 12 regions supported by more than one differential CpG, including the *FAM184B* and *AHRR* genes (Table 4).

In the cancer subset, DMR analysis found one region supported by more than one differential CpG for smokers in contrast to nonsmokers in the initial model corresponding to the *AHRR* gene (Table S4). In the final model, DMR analysis showed only two DMR regions, only the one corresponding *AHRR* supported by more than one differentially methylated CpG (Table 5).

### Gene Prioritization Results and Corresponding DNA Methylation Analysis

The genes found to be differentially methylated in the genome-wide analysis, supported by DMPs and DMRs, were used as training input in the prioritization software; the results of the prioritization in Endeavour showed the following 167 genes significantly related to the training genes: *CDK4*, *TP53*, *CDKN1A*, *CDK6*, *CCND1*, *RB1 CDKN2B*, *CDKN2D*, *CDKN1B*, *CDKN2C*, *CCND2*, *MYC*, *CCND3*, *MDM2*, *E2F1*, *KRAS*, *THBS1*, *ARNT*, *ATM*, *BRCA1*, *ERBB2*, *SIM1*, *CDC6*, *EGFR*, *AKT1*, *CDK2*, *CHEK2*, *TP73*, *MMP2*, *PIK3R1*, *HRAS*, *BCL2*, *PTEN*, *MLH1*, *CCNB1*, *ATR*, *CASP3*, *TGFB1*, *CCNA2*, *SMAD4*, *CDC7*, *CCNH*, *IGF1R*, *ANKRD12*, *NFKB1*, *PCNA*, *RBL1*, *MAPK14*, *E2F3*, *PDGFRA*, *RUNX1*, *SP1*, *SMAD3*, *HIF3A*, *E2F2*, *MYCN*, *RASSF1*, *STAT3*, *MMRN1*, *PLK1*, *MET*, *RELA*, *CCNE1*, *NCOA3*, *HIF1A*, *JUN*, *TFDP1*, *CHEK1*, *SKP2*, *TBRG1*, *PARP1*, *CREBBP*, *COL1A1*, *TGFBR2*, *NFKBIA*, *ABL1*, *FAS*, *PDGFRB*, *E2F4*, *ESR1*, *CASP8*, *NOTCH1*, *TP63*, *CDK7*, *IGF1*, *CDC25A*, *SMAD2*, *PML*, *CCNE2*, *NPM1*, *CTNNB1*, *AHR*, *HDAC1*, *MMP14*, *RAF1*, *SIM2*, *SIRT1*, *BAX*, *PIK3CA*, *CDKN1C*, *MCM5*, *EP300*, *BRCA2*, *APC*, *APAF1*, *COL1A2*, *MAP2K1*, *MCM3*, *MSH2*, *MCM2*, *MAPK1*, *DAPK1*, *CDK1*, *MCM7*, *FASLG*, *PRKCA*, *NCOA2*, *RBL2*, *CREB1*, *TSC1*, *AURKA*, *MCM6*, *TGFBR1*, *MCM4*, *CCNB2*, *PLEKHA8*, *NCL*, *NCOA1*, *BIRC5*, *NRAS*, *TNF*, *ADAMTS12*, *GSK3B*, *NF1*, *GLI2*, *HDAC2*, *AKT2*, *VEGFA*, *MAPK3*, *TERT*, *HIC1*, *MAPK9*, *PIK3CB*, *ADAMTS9*, *ESR2*, *BRAF*, *TFAP2A*, *CCNG1*, *EGR1*, *CEBPB*, *LMNA*, *ORC1*, *ORC6*, *ADAMTS1*, *RET*, *VHL*, *CCNA1*, *VDR*, *TWIST1*, *DHFR*, *FOS*, *MYOD1*, *ORC4*, *IKBKB*, *ADAMTS5*, *CYCS,* and *ADAM12* (20 duplicated registers). In the noncancer epigenome subset, the DNA methylation analysis focused on the prioritized genes revealed three differentially methylated genes, *TFAP2A*, *AHRR,* and *MAPK14*, in addition to *FAM184B* and *ADAMTS2*, which were already present in the results of genome-wide DNA methylation analysis (Table S5). In cancer-diagnosed subjects, two probes corresponding to *AHRR* were found (Table S6); one of them, cg05575921, overlapped with the results in the noncancer subset (Tables S5 and S6).

Furthermore, DMPs of *AHRR*, *ADAMTS2*, and *FAM184* genes passed the sensitivity analysis for cell composition as follows: For the cancer-free subset, cg04450456 for *FAM184B*, cg02599361 for *ADAMTS2*. For the cancer-diagnosed subset, cg05575921 for the *AHRR* gene and two probes cg21566642 and cg05951221 for intergenic regions (Table 6).

## 4. Discussion

The present work determined a genome-wide DNA methylation signature in heavy smokers using available Illumina DNA methylation 450 K data from oral rinse-derived DNA. This is a secondary study that has provided new insights into DNA methylation profiles by focusing the analysis on the search for potential biomarkers of heavy smokers, which differs from the research of previous authors. In complementation of the described approach, we also conducted a directed evaluation of DNA methylation in heavy smokers of genes selected by bioinformatic gene prioritization software, a promising tool not previously used for this question. With this approach, the main results presented here support *AHRR* as an epigenomic biomarker of tobacco smoking in oral rinse but also identified *ADAMTS2* and *FAM184* as new genes involved in tobacco smoking as candidate biomarkers.

The present study was not limited to evaluating each differentially methylated CpG site regardless of its nearness to other CpGs. In contrast, we analyzed the data to detect differential DNA methylation at a regional level, bearing in mind the integration between DMP and DMR analyses, which was not previously achieved. In addition, we performed a combined strategy including (I) a hypothesis-free genome-wide approach for differential methylation analysis and (II) a prioritization approach that integrated the newly discovered genes and the genes previously related to oropharyngeal cancer. Therefore, the identified genes are suggested to be tobacco-related markers that are possibly related to cancer pathogenesis.

### 4.1. AHRR

Here, we found that the *AHRR* gene was differentially hypomethylated at the CpG site corresponding to the Illumina probe cg05575921 (hg19 coordinates chr5:373,378), which also intersected with a genomic region of 589 bp in chromosome 5 identified in our DMR analysis (hg19 coordinates chr5:373299-373887); this region was concordantly hypomethylated in heavy smoker subjects in both subsets of the present study (cancer-free and cancer-diagnosed), indicating the utility of this epigenetic pattern in both subsets. The *AHRR* gene encodes the protein aryl hydrocarbon receptor repressor, which represses the transcriptional activity of the aryl hydrocarbon receptor and mediates dioxin toxicity, xenobiotic metabolism, and the immune response [35,36]. Concordantly, another epigenome-wide association study (EWAS) focused not on oral rinse but on an invasive procedure to analyze solid tissue samples from the oral masticatory mucosa of the hard palate and concluded that hypomethylation of the aryl hydrocarbon receptor repressor *AHRR* is differentially methylated genes in smokers [37]. The concordance of the present result with that reported by Richter et al. demonstrates the possibility of using *AHRR* DNA methylation in smokers in a routine noninvasive screening test in saliva.

### 4.2. ADAMTS2

The present work identified a pattern of *ADAMTS2* differential hypermethylation in heavy smoker subjects in comparison with controls in two neighboring CpGs corresponding to the cg02599361 and cg10208897 Illumina probes in the cancer-free subset (Table 2, Table 6, Tables S1 and S6), located in CpG islands in the gene body of *ADAMTS2*. This gene encodes a protein that cleaves the pro-peptides of collagen and has been implicated in connective tissue disorders and fibrosis (disintegrin-like and metalloproteinase domain with thrombospondin motifs). Interestingly, *ADAMTS2* has been found to be upregulated in cancer [38,39]. Further, this gene was not differentially methylated in the cancer-diagnosed subset; therefore, future studies could explore whether an increase in DNA methylation in the CpG islands of *ADAMTS2* could be adaptative or protective for cancer development in heavy smokers.

### 4.3. FAM184B

FAM184B is a family with sequence similarity 184 member B gene expressed in different tissues, including mononuclear cells and the digestive system, according to GTEx Consortium dataset annotation across human tissues [40]. In the present study, we found that FAM184B was differentially hypermethylated in cancer-free subjects in oral rinse samples (Table 2, Table 4, and Table 6). However, at present, its function has not been clarified. Interestingly, the DNA methylation at two CpGs represented by the probes cg16449012 and cg08644678 (localized in different parts of the gene body) was hypermethylated in newborns of mothers exposed to smoking during their pregnancy [41]. In addition, by evaluating DNA methylation using Sequenom MassARRAY, this gene was reported to be differentially hypermethylated in oral cancer compared to adjacent tissue; unfortunately, the exact differentially methylated probe is not available to identify exact concordances [42]. Interestingly, the *FAM184B* gene was found to be downregulated in striated muscle tissue and therefore possibly involved in skeletal muscle dysfunction, which is a frequent extrapulmonary manifestation in chronic obstructive pulmonary disease [43].

### 4.4. MAPK14 and TFAP2A Genes Found by Gene Prioritization

*MAPK14*, a gene of the mitogen-activated protein kinase family, is involved in processes such as proliferation, differentiation, and transcription regulation [44]. *MAPK14* was significantly differentially hypermethylated even after including the alcohol consumption variable in the model (*p* = 0.00158, 1.58 × 10^−3^, Table 6). The *MAPK14* gene has been related to signaling pathways that act as an immune response against oral pathogens [45]. This gene codes for the *MAPK14* enzyme, which has been identified as a possible regulator of inflammation and is a key component of the tumor microenvironment, as chronic inflammation contributes to the development, progression, and regional metastasis of oral carcinomas [46]. *MAPK14* gene expression seems to be decreased at the transcription level in response to tobacco metabolites in RNAseq, but this was not confirmed by qPCR. Interestingly, *MAPK14* was also found to decrease progressively at higher grades of renal clear cell carcinoma [47].

Finally, the present study found that the *TFAP2A* gene was differentially hypomethylated in heavy smokers compared to controls in the analysis of noncancer subjects (Table S6) but not for the subset of cancer-diagnosed subjects. *TFAP2A* encodes the transcription factor AP-2 alpha protein, which binds specific ADN sequences to activate or inhibit gene expression. Interestingly, in cancer (melanoma cells), it has been related to nasopharyngeal cancer growth [48], and aberrant DNA hypermethylation has been found at its promoter, associated with reduced expression and proposed as a possible target/marker [49]. *MAPK14* and *TFAP2A* genes were not identified in the genomic approach, which considers FDR correction. Instead, these genes were detected through gene prioritization for targeted DNA methylation analysis, demonstrating statistical significance. However, could be important to be included both genes in future studies.

Previous studies reported DNA differentially methylated genes in smoking. Richter et al. [37] reported significant hypomethylation of *AHRR* and *CYP1B1* in the buccal and airway epithelium of smokers. Our study showed concordat differential hypomethylation for the *AHRR* gene (cg04066994) but not for the *CYP1B1* gene, in mouthwashes, with CpG sites evaluated a very low delta of Betas showing <0.05 and with no significant *p*-values (*p* value > 0.5). Considering that the buccal rinse uses different cell types than the biopsies in the cited study [37], only partial coincidences of DNA methylation are expected.

The results from epithelial cells may have significant implications for epithelial cancer. On the other hand, the buccal rinse procedure is noninvasive and could make the *AHRR* marker, more easily usable on tests with personalized medicine applications. In another study on smoking and DNA methylation, Christiansen et al. identified the *SLAMF7* gene as differentially hypomethylated in smokers using blood samples [50]. In our all CpG sites evaluated for *SLAMF7* (cg23844325, cg07837085, cg11721194, cg04244970, and cg04345766 probes) showed a very low delta of betas values (delta of beta <<0.05) with no significant differences between groups (*p*-value > 0.5). This discrepancy may be due to the differences in the tissues evaluated between the two studies.

DNA methylation plays a particular role in cell differentiation and the maintenance of cell specificity. It represents an important mechanism for cells to react to persistent external stimuli, allowing somatic cells to adjust gene expression to a particular environment in a long-term manner that can be passed on to daughter cells [51]. Therefore, the deleterious effects of smoking on tissue integrity are driven by mechanisms such as changes in DNA methylation patterns and reduced expression of repair genes [52]. It is appropriate to test the DNA methylation patterns reported here in the low-cost molecular assays [53].

There are several limitations to the present study. As this investigation is a secondary analysis, we could only access the data included in the Gene Expression Omnibus Repository, and full raw fluorescence data were unavailable. The cell composition analysis in this study differentiates merely two cell types (epithelial cells and leukocytes) and is optimized for children, potentially reducing its effectiveness for adults. Nevertheless, we employed the most suitable and currently available tool to account for cell composition heterogeneity, ensuring reliable results. Lastly, this study does not address transcriptomic analysis due to the lack of expression data, a limitation linked to the mouthwash samples examined in this research.

This study contributes new markers to be verified in future longitudinal studies in oral rinse samples, which are local but noninvasive specimens, promising for future routine screening and implementable for precision/personalized medicine. Identifying altered epigenetic patterns also allows us to focus future studies on identifying strategies to restore or modify gene regulation in the cells of patients for convenience and to handle plastic epigenetic changes/patterns to benefit patient health [54].

## 5. Conclusions

Differential methylation in *FAM184B*, *AHRR*, and *ADAMTS2* genes establish these genes as biomarker candidates in mouthwash samples, relevant for further studies in clinical settings. Analysis targeting these genes and related pathways is warranted.

## Figures and Tables

**Table 1:** Demographic characteristics of the participants corresponding to the DNA methylomes included in the study.

DNA Methylomes from Cancer-Free Subset			
	Heavy Smokers (*n* = 37)	Controls (*n* = 31)	*p*-Values
Age, years (mean ± standard deviation)	62.73 ± 8.044	58.45 ± 12.01	§: 0.097
Males, *n* (%)	24 (64.86%)	19 (61.29)	†: 0.959
Alcohol consumption			‡: 0.041 *
No alcohol consumption, *n* (%)	4 (10.81%)	7 (22.58)	
Alcohol low frequency, *n* (%)	20 (54.05)	21 (67.74)	
Alcohol high frequency, *n* (%)	13(35.14)	3 (9.68)	
**DNA methylomes from cancer-diagnosed subset**			
	**Heavy smokers (*n* = 92)**	**Controls (*n* = 47)**	***p*-values**
Age, years (mean ± standard deviation)	57.57 ± 12.55	60.77 ± 11.07	§: 0.144
Males, *n* (%)	69(76)	22(66)	†: 0.286
Alcohol consumption			‡: 0.002 *
No alcohol consumption, *n* (%)	4 (0.44%)	6 (13%)	
Alcohol low frequency, *n* (%)	42 (45.6%)	31 (67%)	
Alcohol high frequency, *n* (%)	46 (50%)	10 (21%)	

*n* number of subjects. § Student’s *t*-test. † chi-square test. ‡ Fisher’s exact test. * Statistical significance.

**Table 2:** Differentially methylated positions (DMPs) were found in genome-wide analysis with the initial statistical model for heavy smokers in the cancer-free subset.

Probe ID	Gene Symbol	GeneID	logFC	AveExpr	t	*p*-Value	Adj.P.Val	B	Δβ	Dir
cg02599361	*ADAMTS2*	9509	0.133	0.664	4.93	5.8 × 10^−6^	0.026	2.45	0.10	↑
cg04450456	*FAM184B*	27146	0.101	0.811	5.75	2.42 × 10^−7^	0.008	5.57	0.10	↑
cg15017067	*FAM184B*	27146	0.093	0.766	4.65	1.62 × 10^−5^	0.037	1.44	0.09	↑

Note: The initial statistical model included sex and gender variables in addition to comparing heavy smokers and nonsmokers to identify genomic regions with different DNA methylation patterns. Abbreviations: Probe ID: code of Illumina 450 K probe. Adj.P.Val: adjusted *p* value for multiple tests with Benjamini & Hochberg. Gene Symbol: Unique symbol of the NCBI Gene database. B: logarithm of the probability of difference between groups. LogFC: maximum value of relative change (fold change) between smoking and nonsmoking groups. Δβ: Absolute difference between β methylation between heavy smokers and nonsmokers. Dir: direction of DNA methylation difference where **↑** corresponds to differentially hypermethylated positions in heavy smokers.

**Table 3:** Differentially methylated genomic positions in the initial statistical model for heavy smokers in the cancer-diagnosed subset.

Probe ID	Gene	GeneID	AveExpr	t	*p*-Value	Adj.P.Val	B	Δβ	Dir
cg05575921	*AHRR*	57491	0.72	−5.37	3.20 × 10^−7^	2.23 × 10^−2^	4.86	−0.13	↓
cg05951221	-	-	0.40	−7.16	4.32 × 10^−11^	9.05 × 10^−6^	13.63	−0.10	↓
cg06126421		-	0.50	−5.22	6.55 × 10^−7^	3.92 × 10^−2^	4.21	−0.11	↓
cg21566642	-	-	0.40	−6.44	1.81 × 10^−9^	2.53 × 10^−4^	9.95	−0.10	↓

Note: The initial statistical model included sex and gender variables and compared heavy smokers and nonsmokers to identify genomic regions with different DNA methylation patterns. Abbreviations: Probe ID: code of Illumina 450 K probe. Adj.P.Val: adjusted *p* value for multiple tests with Benjamini & Hochberg. Gene Symbol: Unique symbol of the NCBI Gene database. B: logarithm of the probability of difference between groups. LogFC: maximum value of relative change (fold change) between smoking and nonsmoking groups. Δβ: Absolute difference between β methylation between heavy smokers and nonsmokers. Dir: direction of DNA methylation difference where and ↓ corresponds to differentially hypomethylated positions in heavy smokers.

**Table 4:** DMRs identified in genome-wide analysis using the final statistical model for heavy smokers in the cancer-free subset.

hg19 Coordinates	Width	Gene(s)	Group	#p	Minpval	Meanpval	Maxbetafc	Mean Dbeta	Dir
chr12:2943902-2944493	592	*NRIP2*	1st exon, 5′UTR, TSS200	8	2.50 × 10^−8^	3.72 × 10^−8^	0.015	0.066	↑
chr4:17643702-17643749	48	*FAM184B*	Body	2	3.11 × 10^−6^	3.11 × 10^−6^	0.015	0.098	↑
chr2:233215939-233217079	1141			6	2.89 × 10^−3^	6.95 × 10^−3^	0.015	0.071	↑
chr1:102312608-102312671	64	*OLFM3*	Body	3	8.87 × 10^−3^	8.89 × 10^−3^	0.013	0.078	↑
chr19:49001890-49002477	588	*LMTK3*	Body	3	9.16 × 10^−3^	2.16 × 10^−2^	0.012	0.071	↑
chr17:80708279-80708513	235	*FN3K*, *TBCD*	Body, TSS1500	3	1.25 × 10^−2^	1.26 × 10^−2^	0.018	0.093	↑
chr19:18888799-18889003	205	*CRTC1*	3′UTR	2	1.41 × 10^−2^	1.84 × 10^−2^	0.011	0.075	↑
chr5:373299373887	589	*AHRR*	Body	3	1.61 × 10^−2^	2.34 × 10^−2^	−0.019	−0.069	↓
chr1:19110734-19110978	245			3	2.51 × 10^−2^	2.55 × 10^−2^	0.029	0.132	↑
chr7:52341648-52342124	477			3	3.43 × 10^−2^	4.09 × 10^−2^	0.010	0.062	↑
chr21:37437505-37437565	61	*SETD4*	TSS1500	2	4.17 × 10^−2^	4.24 × 10^−2^	−0.025	−0.062	↓
chr4:100242862-100242957	96	*ADH1B*	TSS1500	2	4.23 × 10^−2^	4.50 × 10^−2^	0.013	0.074	↑

Note: The final statistical model included sex, gender, and alcohol consumption level in addition to the comparing heavy smokers and nonsmokers to identify genomic regions with different DNA methylation patterns. Abbreviations. *hg19 coordinates*: coordinates of localization in the human genome hg19, starting with the chromosome number, followed by the coordinates where the differential DNA methylation was found. Width: the width of the genomic region in bp. Gene(s): the corresponding gene(s) associated with the region according to DMRcate’s function. Group: Illumina intergenic position original annotation. #p: Number of probes/CpGs that support the corresponding genomic ranges. minpval: minimum of the *p* value corresponding to neighboring probes/CpGs. meanpval: mean of the *p* value corresponding to neighboring probes/CpGs. maxbetafc: the major fold change M value corresponding to the probes inside the corresponding genomic range. mean Dbeta: the net difference between beta values between groups (HS-control). Dir: direction of DNA methylation difference where **↑** corresponds to differentially hypermethylated regions in heavy smokers, and **↓** corresponds to differentially hypomethylated regions in heavy smokers.

**Table 5:** DMRs were identified in genome-wide analysis with the final statistical model for heavy smokers in the cancer-diagnosed subset.

hg19 Coordinates	Width	Gene(s)	Group	#p	Minpval	Meanpval	Maxbetafc	Mean Dbeta	Dir
chr5:373299-373887	589	*AHRR*	Body	3	7.58 × 10^−5^	3.75 × 10^−3^	−0.02	−0.07	↓
chr19:17000585-17000585	1	*F2RL3*	Body	1	4.50 × 10^−2^	4.50 × 10^−2^	−0.01	−0.08	↓

Note: The final statistical model included sex, gender, and alcohol consumption level in addition to comparing heavy smokers and nonsmokers to identify genomic regions with different DNA methylation patterns. Abbreviations. hg19 coordinates: coordinates of localization in the human genome hg19, starting with the chromosome number, followed by the coordinates where differential DNA methylation was found. Width: the width of the genomic region in bp. Gene(s): the corresponding gene(s) associated with the region according to DMRcate’s function. Group: Illumina intergenic position original annotation. #p: Number of probes/CpGs that support the corresponding genomic ranges. minpval: minimum of the *p* value corresponding to neighboring probes/CpGs. meanpval: mean of the *p* value corresponding to neighboring probes/CpGs. maxbetafc: the major fold change M value corresponding to the probes inside the corresponding genomic range. mean Dbeta: the net difference between beta values between groups (HS-control). Dir: direction of DNA methylation difference where ↓ corresponds to differentially hypomethylated regions in heavy smokers.

**Table 6:** Differentially methylated genes/regions that pass cell composition sensibility analysis.

Gene	ProbeID	logFC	r	t	*p*-Value	Adj.P.Val	B	Δβ	Dir
**Cancer diagnosed subset**									
*FAM184B*	cg04450456	0.10	0.81	5.37	1.16 × 10^−6^	9.24 × 10^−3^	3.98	0.10	↑
*ADAM2*	cg02599361	0.13	0.66	5.34	1.27 × 10^−6^	9.31 × 10^−3^	3.89	0.10	↑
**Cancer-free subset**									
*AHRR*	cg05575921	−0.10	0.72	−5.66	8.89 × 10^−8^	5.31 × 10^−3^	6.07	−0.13	↓
Intergenic	cg21566642	−0.10	0.40	−6.65	6.62 × 10^−10^	1.38 × 10^−4^	10.91	−0.10	↓
Intergenic	cg05951221	−0.10	0.40	−6.12	9.69 × 10^−9^	1.35 × 10^−3^	8.24	−0.10	↓

Note: The sensitivity statistical model included cell composition of leucocytes versus epithelial cells, in addition to sex, gender, and alcohol consumption levels, further supporting the findings. Dir: direction of DNA methylation difference where **↑** corresponds to differentially hypermethylated genes and ↓ corresponds to differentially hypomethylated genes heavy smokers.

## Data Availability

Data used in this study was available in Gene Expression Omnibus data set GSE70977 (available at https://www.ncbi.nlm.nih.gov/geo/ accessed on 1 January 2021).

## Supplementary Materials

The following supporting information can be downloaded at: https://www.mdpi.com/article/10.3390/biomedicines11071797/s1, Table S1: Genomic differentially methylated probes in the final statistical model for heavy smokers, inside the cancer-free subset; Table S2: Genomic differentially methylated probes in the final statistical model for smoking habit, inside the cancer-diagnosed subset; Table S3: Differentially methylated regions in the initial statistical model for smoking habit in the group, inside the cancer-free subset; Table S4: Differentially methylated regions in heavy smokers, genome wide-analysis for the cancer-free subset; Table S5: Differentially methylated probes focused on genes prioritized using the final statistics model; inside the cancer-free subset; Table S6: Differentially methylated probes focused on genes prioritized using the final statistics model; resulting in the cancer-diagnosed subset.

## References

[1] ALHarthi S.S.Y., Natto Z.S., Midle J.B., Gyurko R., O’Neill R., Steffensen B. (2019). Association between time since quitting smoking and periodontitis in former smokers in the National Health and Nutrition Examination Surveys (NHANES) 2009 to 2012. J. Periodontol..

[2] Boyle J.O., Gumus Z.H., Kacker A., Choksi V.L., Bocker J.M., Zhou X.K., Yantiss R.K., Hughes D.B., Du B., Judson B.L. (2010). Effects of cigarette smoke on the human oral mucosal transcriptome. Cancer Prev. Res..

[3] Canova C., Richiardi L., Merletti F., Pentenero M., Gervasio C., Tanturri G., Garzino-Demo P., Pecorari G., Talamini R., Barzan L. (2010). Alcohol, tobacco and genetic susceptibility in relation to cancers of the upper aerodigestive tract in northern Italy. Tumori.

[4] Sharma M.K., Gour N., Pandey A., Wallia D. (2011). Epidemiological study of risk factors for oral, laryngeal and esophageal cancers at a tertiary care hospital in India. Asian Pac. J. Cancer Prev..

[5] Cox L.S., Nollen N.L., Mayo M.S., Faseru B., Greiner A., Ellerbeck E.F., Krebill R., Tyndale R.F., Benowitz N.L., Ahluwalia J.S. (2022). Effect of Varenicline Added to Counseling on Smoking Cessation Among African American Daily Smokers: The Kick It at Swope IV Randomized Clinical Trial. JAMA.

[6] Galan I., Ortiz C., Perez-Rios M., Ayuso-Alvarez A., Rodriguez-Blazquez C., Damian J., Fernandez-Escobar C., Garcia-Esquinas E., Lopez-Cuadrado T. (2023). Light cigarette smoking and all-cause mortality in Spain. A national population-based cohort study. Ann. Epidemiol..

[7] Toporcov T.N., Znaor A., Zhang Z.F., Yu G.P., Winn D.M., Wei Q., Vilensky M., Vaughan T., Thomson P., Talamini R. (2015). Risk factors for head and neck cancer in young adults: A pooled analysis in the INHANCE consortium. Int. J. Epidemiol..

[8] Kawakita D., Hosono S., Ito H., Oze I., Watanabe M., Hanai N., Hasegawa Y., Tajima K., Murakami S., Tanaka H. (2012). Impact of smoking status on clinical outcome in oral cavity cancer patients. Oral. Oncol..

[9] Galvin S., Moran G.P., Healy C.M. (2023). Influence of site and smoking on malignant transformation in the oral cavity: Is the microbiome the missing link?. Front. Oral Health.

[10] Zieba S., Maciejczyk M., Zalewska A. (2021). Ethanol- and Cigarette Smoke-Related Alternations in Oral Redox Homeostasis. Front. Physiol..

[11] Sreekumar V.N. (2019). Global Scenario of Research in Oral Cancer. J. Maxillofac. Oral Surg..

[12] Robles A.I., Harris C.C. (2017). Integration of multiple “OMIC” biomarkers: A precision medicine strategy for lung cancer. Lung. Cancer.

[13] Niedzwiecki M.M., Walker D.I., Vermeulen R., Chadeau-Hyam M., Jones D.P., Miller G.W. (2019). The Exposome: Molecules to Populations. Annu. Rev. Pharmacol. Toxicol..

[14] Vacante M., Borzi A.M., Basile F., Biondi A. (2018). Biomarkers in colorectal cancer: Current clinical utility and future perspectives. World J. Clin. Cases.

[15] Um S.W., Kim Y., Lee B.B., Kim D., Lee K.J., Kim H.K., Han J., Kim H., Shim Y.M., Kim D.H. (2018). Genome-wide analysis of DNA methylation in bronchial washings. Clin. Epigenetics.

[16] Clough E., Barrett T. (2016). The Gene Expression Omnibus Database. Methods Mol. Biol..

[17] Liu H., Chen D., Liu P., Xu S., Lin X., Zeng R. (2019). Secondary analysis of existing microarray data reveals potential gene drivers of cutaneous squamous cell carcinoma. J. Cell Physiol..

[18] Beltran-Garcia J., Osca-Verdegal R., Mena-Molla S., Garcia-Gimenez J.L. (2019). Epigenetic IVD Tests for Personalized Precision Medicine in Cancer. Front. Genet..

[19] Joehanes R., Just A.C., Marioni R.E., Pilling L.C., Reynolds L.M., Mandaviya P.R., Guan W., Xu T., Elks C.E., Aslibekyan S. (2016). Epigenetic Signatures of Cigarette Smoking. Circ. Cardiovasc. Genet..

[20] Lee M.K., Hong Y., Kim S.Y., London S.J., Kim W.J. (2016). DNA methylation and smoking in Korean adults: Epigenome-wide association study. Clin. Epigenetics.

[21] Langevin S.M., Eliot M., Butler R.A., Cheong A., Zhang X., McClean M.D., Koestler D.C., Kelsey K.T. (2015). CpG island methylation profile in non-invasive oral rinse samples is predictive of oral and pharyngeal carcinoma. Clin. Epigenetics.

[22] Barrett T., Wilhite S.E., Ledoux P., Evangelista C., Kim I.F., Tomashevsky M., Marshall K.A., Phillippy K.H., Sherman P.M., Holko M. (2013). NCBI GEO: Archive for functional genomics data sets--update. Nucleic Acids Res..

[23] Ikeda S., Kang M.-I., Ohtake F., Ikeda S., Kato H.K., Ohtake F., Tsutsui Y. (2016). Hyperbolic Discounting, the Sign Effect, and the Body Mass Index. Behavioral Economics of Preferences, Choices, and Happiness.

[24] Davis S., Meltzer P.S. (2007). GEOquery: A bridge between the Gene Expression Omnibus (GEO) and BioConductor. Bioinformatics.

[25] Fortin J.P., Labbe A., Lemire M., Zanke B.W., Hudson T.J., Fertig E.J., Greenwood C.M., Hansen K.D. (2014). Functional normalization of 450k methylation array data improves replication in large cancer studies. Genome Biol..

[26] Gentleman R.C., Carey V.J., Bates D.M., Bolstad B., Dettling M., Dudoit S., Ellis B., Gautier L., Ge Y., Gentry J. (2004). Bioconductor: Open software development for computational biology and bioinformatics. Genome Biol..

[27] Ivorra C., Fraga M.F., Bayon G.F., Fernandez A.F., Garcia-Vicent C., Chaves F.J., Redon J., Lurbe E. (2015). DNA methylation patterns in newborns exposed to tobacco in utero. J. Transl. Med..

[28] Hernandez H.G., Sandoval-Hernandez A.G., Garrido-Gil P., Labandeira-Garcia J.L., Zelaya M.V., Bayon G.F., Fernandez A.F., Fraga M.F., Arboleda G., Arboleda H. (2018). Alzheimer’s disease DNA methylome of pyramidal layers in frontal cortex: Laser-assisted microdissection study. Epigenomics.

[29] Peters T.J., Buckley M.J., Statham A.L., Pidsley R., Samaras K., Lord R.V., Clark S.J., Molloy P.L. (2015). De novo identification of differentially methylated regions in the human genome. Epigenetics Chromatin.

[30] Hernandez H.G., Hernandez-Castaneda A.A., Pieschacon M.P., Arboleda H. (2021). ZNF718, HOXA4, and ZFP57 are differentially methylated in periodontitis in comparison with periodontal health: Epigenome-wide DNA methylation pilot study. J. Periodontal. Res..

[31] Carnielli C.M., Macedo C.C.S., De Rossi T., Granato D.C., Rivera C., Domingues R.R., Pauletti B.A., Yokoo S., Heberle H., Busso-Lopes A.F. (2018). Combining discovery and targeted proteomics reveals a prognostic signature in oral cancer. Nat. Commun..

[32] Tranchevent L.C., Ardeshirdavani A., ElShal S., Alcaide D., Aerts J., Auboeuf D., Moreau Y. (2016). Candidate gene prioritization with Endeavour. Nucleic Acids Res..

[33] Middleton L.Y.M., Dou J., Fisher J., Heiss J.A., Nguyen V.K., Just A.C., Faul J., Ware E.B., Mitchell C., Colacino J.A. (2022). Saliva cell type DNA methylation reference panel for epidemiological studies in children. Epigenetics.

[34] Fisher J., Middleton L., Bakulski K. BeadSorted.Saliva.EPIC: Illumina DNA Methylation Data on Sorted Saliva Cell Populations. R Package. https://bioconductor.org/packages/devel/data/experiment/vignettes/BeadSorted.Saliva.EPIC/inst/doc/BeadSorted.Saliva.EPIC.html.

[35] Yang S.Y., Ahmed S., Satheesh S.V., Matthews J. (2018). Genome-wide mapping and analysis of aryl hydrocarbon receptor (AHR)- and aryl hydrocarbon receptor repressor (AHRR)-binding sites in human breast cancer cells. Arch. Toxicol..

[36] Vogel C.F.A., Haarmann-Stemmann T. (2017). The aryl hydrocarbon receptor repressor—More than a simple feedback inhibitor of AhR signaling: Clues for its role in inflammation and cancer. Curr. Opin. Toxicol..

[37] Richter G.M., Kruppa J., Munz M., Wiehe R., Hasler R., Franke A., Martins O., Jockel-Schneider Y., Bruckmann C., Dommisch H. (2019). A combined epigenome- and transcriptome-wide association study of the oral masticatory mucosa assigns CYP1B1 a central role for epithelial health in smokers. Clin. Epigenetics.

[38] Su S.C., Hsieh M.J., Liu Y.F., Chou Y.E., Lin C.W., Yang S.F. (2016). ADAMTS14 Gene Polymorphism and Environmental Risk in the Development of Oral Cancer. PLoS ONE.

[39] Kumar S., Rao N., Ge R. (2012). Emerging Roles of ADAMTSs in Angiogenesis and Cancer. Cancers.

[40] Aguet F., Anand S., Ardlie K.G., Gabriel S., Getz G.A., Graubert A., Hadley K., Handsaker R.E., Huang K.H., Kashin S. (2020). The GTEx Consortium atlas of genetic regulatory effects across human tissues. Science.

[41] Wiklund P., Karhunen V., Richmond R.C., Parmar P., Rodriguez A., De Silva M., Wielscher M., Rezwan F.I., Richardson T.G., Veijola J. (2019). DNA methylation links prenatal smoking exposure to later life health outcomes in offspring. Clin. Epigenetics.

[42] Khongsti S., Lamare F.A., Shunyu N.B., Ghosh S., Maitra A., Ghosh S. (2018). Whole genome DNA methylation profiling of oral cancer in ethnic population of Meghalaya, North East India reveals novel genes. Genomics.

[43] Willis-Owen S.A.G., Thompson A., Kemp P.R., Polkey M.I., Cookson W., Moffatt M.F., Natanek S.A. (2018). COPD is accompanied by co-ordinated transcriptional perturbation in the quadriceps affecting the mitochondria and extracellular matrix. Sci. Rep..

[44] NCBI (2022). MAPK14 Mitogen-Activated Protein Kinase 14 [Homo Sapiens (Human)]—Gene. https://www.ncbi.nlm.nih.gov/gene?Db=gene&Cmd=DetailsSearch&Term=1432.

[45] Groeger S., Jarzina F., Domann E., Meyle J. (2017). Porphyromonas gingivalis activates NFkappaB and MAPK pathways in human oral epithelial cells. BMC Immunol..

[46] Li Z., Liu F.Y., Kirkwood K.L. (2020). The p38/MKP-1 signaling axis in oral cancer: Impact of tumor-associated macrophages. Oral Oncol..

[47] Huang Y., Wang Q., Tang Y., Liu Z., Sun G., Lu Z., Chen Y. (2022). Identification and validation of a cigarette smoke-related five-gene signature as a prognostic biomarker in kidney renal clear cell carcinoma. Sci. Rep..

[48] Shi D., Xie F., Zhang Y., Tian Y., Chen W., Fu L., Wang J., Guo W., Kang T., Huang W. (2014). TFAP2A regulates nasopharyngeal carcinoma growth and survival by targeting HIF-1alpha signaling pathway. Cancer Prev. Res..

[49] Hallberg A.R., Vorrink S.U., Hudachek D.R., Cramer-Morales K., Milhem M.M., Cornell R.A., Domann F.E. (2014). Aberrant CpG methylation of the TFAP2A gene constitutes a mechanism for loss of TFAP2A expression in human metastatic melanoma. Epigenetics.

[50] Christiansen C., Castillo-Fernandez J.E., Domingo-Relloso A., Zhao W., El-Sayed Moustafa J.S., Tsai P.C., Maddock J., Haack K., Cole S.A., Kardia S.L.R. (2021). Novel DNA methylation signatures of tobacco smoking with trans-ethnic effects. Clin. Epigenetics.

[51] De Oliveira S.R., Da Silva I.C., Mariz B.A., Pereira A.M., De Oliveira N.F. (2015). DNA methylation analysis of cancer-related genes in oral epithelial cells of healthy smokers. Arch. Oral Biol..

[52] Carta C.F.L., Oliveira Alves M.G., de Barros P.P., Campos M.S., Scholz J., Jorge A.O.C., Nunes F.D., Almeida J.D. (2018). Screening methylation of DNA repair genes in the oral mucosa of chronic smokers. Arch. Oral Biol..

[53] Pinzon-Reyes E., Alvarez W.A., Rondon-Villarreal P., Hernandez H.G. (2020). Softepigen: Primers Design Web-Based Tool for MS-HRM Technique. IEEE/ACM Trans. Comput. Biol. Bioinform..

[54] Flavahan W.A. (2020). Epigenetic plasticity, selection, and tumorigenesis. Biochem. Soc. Trans..

