# Supplementary material for: Aberrant AHRR, ADAMTS2 and FAM184 DNA Methylation: Candidate Biomarkers in the Oral Rinse of Heavy Smokers
Source: Biomedicines. 2023 Jun 23;11(7):1797. doi: 10.3390/biomedicines11071797 (PMC10376800; doi:10.3390/biomedicines11071797)
Supplement: Supplementary file 1 [file biomedicines-11-01797-s001.zip › biomedicines-2328564-supplementary.pdf]

SUPPLEMENTARY TABLES

Table S1. Genomic differentially methylated probes found in genome-wide analysis with the final statistical model for heavy smokers in the cancer-free subset.

Probe ID	Gene Symbol	logFC	AveExpr	T	P.Value	Adj.P.Val	B	Δβ	Dir
**cg02599361**	*ADAMTS2*	0.136	0.664	5.70	3.13E-07	0.012	5.37	0.10	↑
**cg04450456**	*FAM184B*	0.097	0.811	4.78	1.06E-05	0.038	1.88	0.10	↑

Note: The final statistical model included sex, age, and alcohol consumption level, in addition to the comparison between heavy smokers and nonsmokers to find genomic regions with different DNA methylation patterns. Abbreviations: Probe ID: code of Illumina 450K probe. Adj.P.Val: adjusted p value for multiple tests with Benjamini & Hochberg. Gene Symbol: Unique symbol of the NCBI Gene database. B: logarithm of the probability of difference between groups. LogFC: maximum value of relative change (fold change) between smoking and nonsmoking groups. Δβ: Absolute difference between β methylation between heavy smokers and nonsmokers. Dir: direction of DNA methylation difference where ↑ corresponds to differentially hypermethylated positions in heavy smokers.

Table S2. Genomic differentially methylated probes in the final statistical model for smoking habit, inside the cancer-diagnosed subset.

Probe ID	Gene Symbol	logFC	AveExpr	T	P.Value	Adj.P.Val	B	Δβ	Dir
**cg05951221**	Intergenic	-0.11	0.40	-7.16	4.32E-11	9.05E-06	13.63	-0.10	↓
**cg21566642**	Intergenic	-0.11	0.40	-6.44	1.81E-09	2.53E-04	9.95	-0.10	↓

Note: The final statistical model included sex, age, and alcohol consumption level, in addition to the comparison between heavy smokers and nonsmokers to find genomic regions with different DNA methylation patterns. Abbreviations: Probe ID: code of Illumina 450K probe. Adj.P.Val: adjusted p value for multiple tests with Benjamini & Hochberg. Gene Symbol: Unique symbol of the NCBI Gene database. B: logarithm of the probability of difference between groups. LogFC: maximum value of relative change (fold change) between smoking and nonsmoking groups. Δβ: Absolute difference between β methylation between heavy smokers and nonsmokers. Dir: direction of DNA methylation difference where ↓ corresponds to differentially hypomethylated positions in heavy smokers.

Table S3. Differentially methylated regions in the initial statistical model for heavy smokers, in the cancer-free subset.

hg19 coordinates	width	Gene(s)	Group	#p	Minpval	meanpval	maxbetafc	mean Dbet	Dir
**chr12:**2943902-2944493	592	*NRIP2*	1stExon, 5'UTR, TSS200, TSS1500	8	8.65E-11	1.81E-10	0.016	0.07	↑
chr4:17643702-17643749	48	*FAM184B*	Body	2	1.75E-8	1.76E-8	0.016	0.10	↑
chr1:102312608-102312671	64	*OLFM3*	Body	3	1.10E-4	1.11E-4	0.015	0.08	↑
chr20:44838776-44839734	959	*CDH22*	Body	4	2.32E-4	8.38E-3	0.014	0.06	↑
chr14:34268920-34270437	1518	*NPAS3*	Body,3'UTR	7	3.13E-4	6.95E-3	0.016	0.06	↑
chr19:49000743-49002477	1735	*LMTK3*	Body	6	4.01E-4	2.03E-2	0.013	0.07	↑
chr19:18888081-18889003	923	*CRTC1*	Body,3'UTR	3	4.60E-4	4.39E-3	0.013	0.07	↑
chr7:52341469-52342124	656			4	5.55E-4	7.99E-4	0.012	0.06	↑
chr7:27231491-27232150	660			4	4.91E-3	1.02E-2	-0.015	-0.06	↓
chr3:22412124-22412746	623			3	5.07E-3	6.71E-3	0.021	0.11	↑
chr10:1405937-1406508	572	*ADARB2*	Body	2	5.41E-3	9.65E-3	0.015	0.07	↑
chr1:19110734-19110978	245			3	5.72E-3	5.80E-3	0.031	0.13	↑
chr17:80708279-80708513	235	*FN3K, TBCD*	Body, TSS1500	3	6.27E-3	6.73E-3	0.018	0.09	↑
chr4:100242862-100242957	96	*ADH1B*	TSS1500	2	6.78E-3	7.41E-3	0.014	0.07	↑
chr8:144809598-144810339	742	*FAM83H*	Body	4	1.36E-2	2.17E-2	0.010	0.06	↑
chr8:105379726-105379985	260			2	2.24E-2	3.41E-2	0.012	0.06	↑
chr1:211652276-211652688	413	*RD3*	3'UTR,Body	3	3.00E-2	3.13E-2	0.020	0.08	↑
chr8:145755756-145755974	219	*MGC70857 KIAA1688*	TSS1500, 3'UTR,Body	3	3.49E-2	4.30E-2	0.012	0.07	↑

Note: The initial statistical model included sex and gender variables in addition to the comparison between heavy smokers and nonsmokers to find genomic regions with different DNA methylation patterns Abbreviations. *hg19 coordinates:* coordinates of localization in the human genome hg19, starting with the chromosome number, followed by the coordinates where differential DNA methylation was found. Width: the width of the genomic region in bp. Gene(s): the corresponding gene(s) associated with the region according to DMRcate's function. Group: Illumina intergenic position original annotation. #p: Number of probes/CpGs that support the corresponding genomic ranges. minpval: minimum of the p value corresponding to neighboring probes/CpGs. meanpval: mean of the p value corresponding to neighboring probes/CpGs. maxbetafc: the major fold change M value corresponding to the probes inside the corresponding genomic range. **mean Dbeta**: the net difference between beta values between groups (HS-control). **Dir**: direction of DNA methylation difference where ↑ corresponds to differentially hypermethylated positions in heavy smokers, and ↓ corresponds to differentially hypomethylated positions in heavy smokers.

Table S4. Differentially methylated regions in heavy smokers, genome-wide analysis for the cancer-free subset

hg19 coordinates	width	Gene(s)	Group	#p	minpval	meanpval	maxbetafc	mean Dbet	Dir
chr5:373299-373887	589	***AHRR***	Body	3	4.60E-07	5.54E-04	-0.02	-0.07	↓
chr14:106092151-106092151	1			1	2.55E-02	2.55E-02	-0.01	-0.08	↓

Note: The initial statistical model included sex and gender variables in addition to the comparison between heavy smokers and nonsmokers to find genomic regions with different DNA methylation patterns Abbreviations. *hg19 coordinates:* coordinates of localization in the human genome hg19, starting with the chromosome number, followed by the coordinates where differential DNA methylation was found. Width: the width of the genomic region in bp. Gene(s): the corresponding gene(s) associated with the region according to DMRcate's function. Group: Illumina intergenic position original annotation. #p: Number of probes/CpGs that support the corresponding genomic ranges. minpval: minimum of the p value corresponding to neighboring probes/CpGs. meanpval: mean of the p value corresponding to neighboring probes/CpGs. maxbetafc: the major fold change M value corresponding to the probes inside the corresponding genomic range. mean Dbeta: the net difference between beta values between groups (HS-control). Dir: direction of DNA methylation difference where ↑ corresponds to differentially hypermethylated regions in heavy smokers, and ↓ corresponds to differentially hypomethylated regions in heavy smokers.

Table S5. Differentially methylated probes focused on genes prioritized using the final statistical model; inside the cancer-free subset.

Probe ID	Gene Symbol	logFC	AveExpr	t	P.Value	B	Δβ	Dir
cg00326464	*TFAP2A*	-0.06	0.17	-2.76	7.55E-03	-4.41	-0.06	↓
cg02599361	*ADAMTS2*	0.13	0.66	4.93	5.80E-06	2.45	0.10	↑
cg04450456	*FAM184B*	0.10	0.81	5.75	2.42E-07	5.56	0.10	↑
cg05575921	*AHRR*	-0.11	0.72	-3.35	1.33E-03	-2.79	-0.12	↓
cg10208897	*ADAMTS2*	0.09	0.77	4.63	1.75E-05	1.38	0.07	↑
cg15017067	*FAM184B*	0.09	0.77	4.65	1.62E-05	1.44	0.09	↑
cg20559215	*MAPK14*	0.02	0.87	3.21	2.05E-03	-3.13	0.07	↑

[a] Note: the initial statistical model included sex and gender variables, in addition to the contrast between heavy smokers and non-smokers to find differentially methylated positions, in the prioritized genes. Abbreviations: Probe ID: code of Illumina 450K probe. Adj.P.Val: adjusted p value with for multiple tests of Benjamini & Hochberg. Gene Symbol: Unique symbol of the NCBI Gene database. B: logarithm of a probability of difference between groups. LogFC: maximum value of relative change (fold change) between smoking and non-smoking groups. Δβ: Absolute difference between β methylation between heavy smokers and non-smokers. Dir: direction of DNA methylation difference where ↑ corresponds to differentially hypermethylated positions in heavy smokers, and ↓ corresponds to differentially hypomethylated positions in heavy smokers.

Table S6. Differentially methylated probes focused on genes prioritized using the final statistical model; results in the cancer-diagnosed subset.

Probe ID	Gene Symbol	logFC	AveExpr	t	P.Value	B	Δβ	B.nf	B.f	Dir
**cg05575921**	*AHRR*	-0.11	0.72	-4.68	6.76E-6	1.92	-0.13	0.67	0.80	↓
**cg23576855**	*AHRR*	-0.08	0.60	-2.99	3.34E-3	-3.94	-0.09	0.57	0.66	↓

Note I: only statistically sifnificative results are shown (p value <0.005). Note II: the final statistical model included sex and gender variables, alcohol consumption level in addition to the contrast between heavy smokers and non-smoker. Abbreviations: Probe ID: code of Illumina 450K probe. Adj.P.Val: adjusted p value with for multiple tests of Benjamini & Hochberg. Gene Symbol: Unique symbol of the NCBI Gene database. B: logarithm of a probability of difference between groups. LogFC: maximum value of relative change (fold change) between smoking and non-smoking groups. Δβ: Absolute difference between β methylation between heavy smokers and non-smokers. Dir: direction of DNA methylation difference where ↓ corresponds to differentially hypomethylated positions in heavy smokers.
